# Clinical Relevance of Absolute BK Polyoma Viral Load Kinetics in Patients With Biopsy Proven BK Polyomavirus Associated Nephropathy

**DOI:** 10.3389/fmed.2021.791087

**Published:** 2022-01-06

**Authors:** Haris Omić, Johannes Phillip Kläger, Harald Herkner, Stephan W. Aberle, Heinz Regele, Lukas Weseslindtner, Tarek Arno Schrag, Gregor Bond, Katharina Hohenstein, Bruno Watschinger, Johannes Werzowa, Robert Strassl, Michael Eder, Željko Kikić

**Affiliations:** ^1^Division of Nephrology and Dialysis, Department of Medicine III, Medical University of Vienna, Vienna, Austria; ^2^Department of Pathology, Medical University of Vienna, Vienna, Austria; ^3^Department of Emergency Medicine, Medical University of Vienna, Vienna, Austria; ^4^Center for Virology, Medical University of Vienna, Vienna, Austria; ^5^Department of Orthopedics and Trauma Surgery at the Medical University of Vienna in the General Hospital, Vienna, Austria; ^6^Ludwig Boltzmann Institute of Osteology at the Hanusch Hospital of WGKK and AUVA Trauma Centre Meidling, 1st Medical Department, Hanusch Hospital, Vienna, Austria; ^7^Division of Clinical Virology, Department of Laboratory Medicine, Medical University of Vienna, Vienna, Austria; ^8^Department of Urology, Medical University of Vienna, Vienna, Austria

**Keywords:** polyomavirus nephropathy, viral load, viral kinetic, graft survival, renal transplantation

## Abstract

**Introduction:** The absolute BK viral load is an important diagnostic surrogate for BK polyomavirus associated nephropathy (PyVAN) after renal transplant (KTX) and serial assessment of BK viremia is recommended. However, there is no data indicating which particular viral load change, i.e., absolute vs. relative viral load changes (copies/ml; percentage of the preceding viremia) is associated with worse renal graft outcomes.

**Materials and Methods:** In this retrospective study of 91 biopsy proven PyVAN, we analyzed the interplay of exposure time, absolute and relative viral load kinetics, baseline risk, and treatment strategies as risk factors for graft loss after 2 years using a multivariable Poisson-model.

**Results:** We compared two major treatment strategies: standardized immunosuppression (IS) reduction (*n* = 53) and leflunomide (*n* = 30). The median viral load at the index biopsy was 2.15E+04 copies/ml (interquartile range [IQR] 1.70E+03–1.77E+05) and median peak viremia was 3.6E+04 copies/ml (IQR 2.7E+03–3.3E+05). Treatment strategies and IS-levels were not related to graft loss. After correction for baseline viral load and estimated glomerular filtration rate (eGFR), absolute viral load decrease/unit remained an independent risk factor for graft loss [incidence rate ratios [IRR] = 0.77, (95% *CI* 0.61–0.96), *p* = 0.02].

**Conclusion:** This study provides evidence for the prognostic importance of absolute BK viremia kinetics as a dynamic parameter indicating short-term graft survival independently of other established risk factors.

## Introduction

The viral reactivation of BK Virus in an immunocompromised patient may induce BK polyoma virus associated nephropathy (PyVAN) as a serious complication following renal transplantation. PyVAN has a prevalence of 1–10% (1–3). The hallmarks of the diagnosis are the quantitative detection of BKPyV-DNAemia in blood and urine *via* PCR (4, 5), as well as distinct histological and immunohistochemical findings in the renal biopsy as a gold standard for organ invasive infection (6). The histomorphological phenotype of PyVAN is characterized by tubulointerstitial nephritis including the detection of virus-infected tubular epithelial cells by immunohistochemical staining using BK large T-antigen raised against SV40 (7). A more recent diagnostic option is gene expression analysis from biopsy to distinguish PyVAN from T-cell-mediated rejection (TCMR) (8). Untreated PyVAN can lead to progressive graft damage and presents clinically in the form of an asymptomatic deterioration of graft function causing graft failure in up to 10–30% of the patients (3, 9–11).

The absolute viral load is an important diagnostic surrogate for “presumptive PyVAN” (BKPyV load of 10E4 in blood, without biopsy confirmation). While serial assessment of BKPyV viremia after kidney transplantation (KTX) is recommended (12), none of the previously published studies could address serial assessment of viral load kinetics as a risk factor for worse outcomes, mostly because of limited sample-size (13–15). Additionally, complete viral clearance is considered as a treatment success, with however, limited suitability for treatment guidance: the median time to reach complete viral clearance is up to 9 months with a high proportion of patients never achieving this goal (range 25–76%) (16–18).

This underlines the necessity of further solid virological parameters indicating response during treatment. Continuous assessment of BKPyV viremia may be promising, however, there is no data indicating which particular viral load change, i.e., absolute viral load decrease (in copies/ml) vs. relative viral load changes (as percentage of the preceding viremia) is associated with worse renal graft outcomes.

The risk of graft loss and deterioration of graft function may be further influenced by distinct treatment strategies. Currently, the optimal treatment strategy of PyVAN is unknown. The main recommended pillar of treatment remains the reduction of the immunosuppression (IS) (12, 19–21). Reduction of IS includes reduction of calcineurin inhibitors [CNI, Tacrolimus and Cyclosporin A (CyA)] and the reduction or discontinuation of mycophenolate mofetil (MMF) (21). The increased probability of graft rejection associated with decreased immunosuppression necessitates the careful monitoring of renal function and a low biopsy indication threshold (22). Several treatments with antiviral agents, such as leflunomide (a disease modifying drug with immunoregulatory features used to treat different types of rheumatic conditions) (23, 24), cidofovir (25, 26), fluoroquinolones (27), and immunoglobulin therapy (intravenous immunoglobulins [IVIG]) (28, 29) with variable results were attempted.

In this large single-center study of 91 patients with biopsy proven PyVAN, we aimed to analyze the interplay of baseline risk, BK viral load dynamics (absolute and relative changes), and treatment strategies on graft survival after 2 years. By analyzing the kinetics of BK viremia after diagnosis, we aimed at identifying patients under higher risk of graft loss in relation to absolute viral load and relative viral load changes. Serial measurements of BK viral load, graft function, and IS level enabled detailed assessment of graft loss risk using time dependent multivariable models.

## Materials and Methods

### Study Design

**Figure 1:**
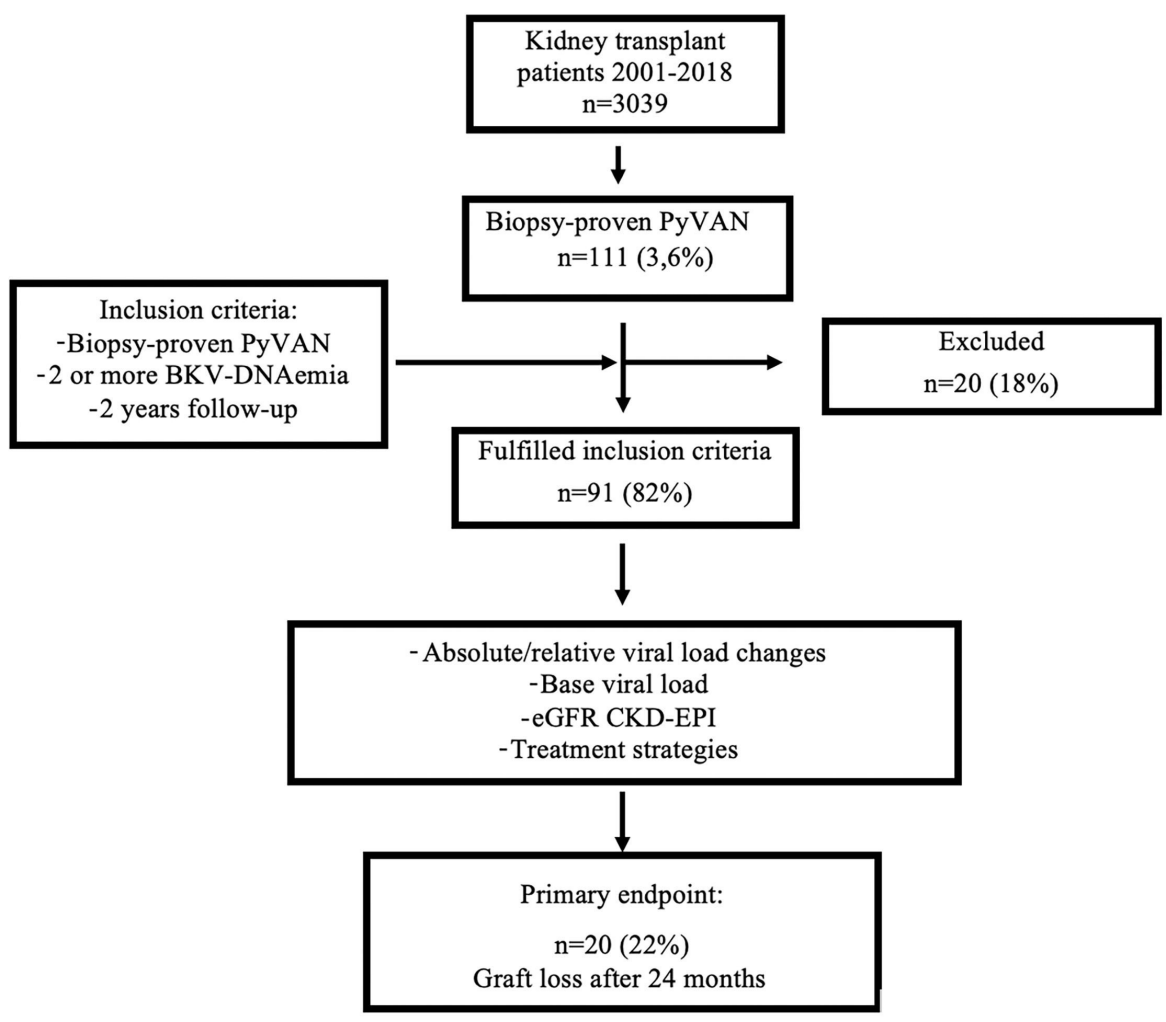
Flowchart. Displays the flow-chart of the study; PyVAN, BK polyomavirus nephropathy; eGFR-CKD-EPI, estimated glomerular filtration rate measured by the CKD-EPI equation; BKV, BK-Virus.

In this retrospective single-center cohort study, all renal transplant recipients, transplanted between 2001 and 2018 at the Medical University of Vienna with biopsy proven PyVAN were considered eligible for study (*N* = 111; 3.6% of all Tx, *N* = 3,039). Following criteria were applied for study inclusion: (a) all patients with biopsy proven PyVAN between 2001 and 2018 supported by compatible histopathological findings and immunohistochemical staining of SV40; (b) two or more positive BK virus- PCR findings during the post-transplantation (TX) period (serial assessment of BK Virus *via* PCR was introduced at 2001); and (c) a follow-up of at least 2 years after index biopsy. This study aimed to assess the association of absolute and relative BK viral load changes over time with transplant survival in the 24 months after the diagnosis of PyVAN and the potential difference in the relation to baseline risk and treatment strategies. The primary outcome was death censored graft-loss, defined as initiation of any renal replacement therapy. The flowchart of the study is shown in Figure 1.

### Parameters and Clinical Findings

This study was approved from the Medical University of Vienna Institutional Review Board (Nr:1291/2020), Vienna, Austria, assuring adherence to the declarations of Helsinki and Istanbul.

Data were assessed retrospectively using electronic and archived medical records. We included following variables: (a) baseline demographic and transplant-associated data (age, gender, number of previous transplants, and donor type [living vs. deceased]), underlying renal disease, number of human leukocyte antigen (HLA) mismatches (0–6), and cold ischemia time (hours), (b) immunosuppression regimen before and after diagnosis of the PyVAN and the trough level of calcineurin inhibitor (Tacrolimus, CyA), (c) viral load in plasma (copies/ml measured by PCR) at months 0, 1, 3, 6, 9, 12, and 24 after PyVAN diagnosis, (d) graft function at diagnosis of PyVAN (estimated glomerular filtration rate measured by the CKD-EPI equation [eGFR CKD-EPI] in ml/min/1.73 m^2^ (30)) as well as at months 1, 3, 6, 9, 12, and 24 after PyVAN diagnosis, (e) date of graft loss, and (f) BANFF single lesions at the time of PyVAN diagnosis and rejection diagnosis. The eGFR-slopes were used to visualize graft function on a longitudinal scale over the follow-up period.

### Treatment of PyVAN and Degree of Immunosuppression

Standard of care for the treatment of PyVAN consisted of two major strategies: (a) a standardized reduction of CNI and/or MMF or (b) switching from MMF or Azathioprine to Leflunomide. After PyVAN diagnosis, the dose of MMF/Azathioprine was either reduced by half or discontinued. In a second step, the dose of the administered CNI was reduced (Tacrolimus levels were targeted to <6 ng/ml, CyA <150 ng/ml). Leflunomide was administered by a daily dose of 20–40 mg/day. Cidofovir or IVIG were used as rescue treatments in rare cases. After transplantation, administration of corticosteroids (or equivalent dose of dexamethasone) was standardized according to our centers protocol with 250 mg on day 1, 125 mg on day 2, 50 mg for days 3–7, 25 mg for days 8–15, 10 mg for days 16–30, and 5 mg further on.

To analyze the effects of the levels of Tacrolimus and CyA at each time-point (months 1, 3, 6, 9, 12, and 24 after the biopsy), we scaled the level of CNI in three categories (low, medium, and high level of CNI exposure), according to the trough levels. Categories were defined as following: low (ng/ml): Tacrolimus 3–5, CyA <40; medium (ng/ml): Tacrolimus 5–7, CyA 40–80; and high (ng/ml): Tacrolimus > 7, CyA > 80.

### Biopsy

Biopsies were performed upon unexplainable graft dysfunction and/or proteinuria (as a standard procedure of post-TX care). None of the included cases originated from protocol biopsies which have been introduced at our center in June 2017. BK viremia without impairment of graft function was not considered as an indication for biopsy. Histopathologic findings were evaluated on formalin-fixed paraffin-embedded sections applying standard methodology. The biopsies were examined for histological signs of viral infection, such as intranuclear inclusions, cellular atypia, tubular epithelial cell degeneration, with rounding, detachment, and cell-apoptosis, and immunohistochemical staining for SV40 large T-antigen (2, 17). Diagnosis and single lesions were scored according to Clasification of Rejection (BANFF) criteria at the time of diagnosis (31). All rejections were treated according to a center-specific protocol with either pulse of steroids or thymoglobulin as described previously (17).

### Dynamics of BK Viremia

Screening for BK infection was performed by testing for BK viremia every 3 months during the first year after TX. Serum viral load was recorded at diagnosis (PyVAN biopsy), at months 1, 3, 6, 9, 12, and 24 after the biopsy as well as months 1, 2, and 3 before the biopsy. Real time PCR was performed by DNA isolation from 200 μl of plasma using the automatic extractor NucliSens EasyMag (bioMérieux, Marcy l'Etoile, France) and eluted to a final installment of 70 μl. BK-polymavirus was quantified using Taqman PCR in real-time with primers and samples inside the small capsid protein VP3 (32). Complete BK viral load clearance was defined as a reduction of viral load under the detection level (<70 copies/ml) (33).

### Statistical Analysis

The research was conducted using a pre-designed model of data collection and all information were inserted into Excel and consequently transferred to the SPSS and Stata analysis system (SPSS: An IBM Company, IBM Corporation, Armonk, NY, USA; Stata 16 Stata Corp, College Station, TX, USA). We present continuous data as mean ± SD, categorized data as absolute count with the relative frequency. To test the null hypothesis of no difference, we used a *t*-test with normally distributed continuous data, Mann–Whitney *U*-test for analysis with no normal distribution. For categorized data, we used the Fisher's exact test. Generally, a two-sided *p* < 0.05 was considered statistically significant. Values from clinical and demographic data were randomly missing only in rare cases and were not included in the statistical analyses.

### Poisson-Regression

To assess the effects of the viral load changes on the graft loss as the outcome of interest, we used the Poisson-regression model. Poisson regression, a form of generalized linear regression, is well-suited to model event rates if the exposure time (offset) for each observed individual is known and matters. The exponentiated regression coefficient equals the incidence rate ratio (*IRR*), which quantifies the effect of a covariable on the event rate. Event rates for graft loss are presented as rate per 100 patient months. We therefore used multivariable Poisson models to estimate *IRR*s with 95% *CI*s of several covariables. As the main exposure covariable, we used changes in viral load (differences between time intervals of the naturally log-transformed plasma viral loads). Other covariables were chosen based on clinical considerations in the counterfactual framework, meaning that they needed to be common causes for changes in viral load and graft loss (baseline eGFR, baseline viral load, levels of the immunosuppression over time, and PyVAN therapy). Individuals may have had several periods of different exposure-levels (viral load), resulting in a panel-design. We included exposure times (offset) for each observation period, which were allowed to differ between individuals and observation periods in our models. Using the patient as the panel identifier, we allowed for the panel design by using random effect models or cluster robust estimators if random effect models could not be computed. We used the Wald-test to test the null hypothesis of *IRR* = 1, meaning no effect of a covariable on graft loss rate.

## Results

### Study Cohort

Of 111 biopsied patients with PyVAN, 20 patients did not fulfill the inclusion criteria. In total 91 patients were included in the final analysis with a mean follow-up after diagnosis of 646 ± 193 days. Most patients were men (*N* = 63; 69.3%) and received their first kidney transplant (*N* = 75; 82%). Deceased donor was the most common type of transplantation (*N* = 74; 81%). Mean recipient age was 51 ± 15 years. As shown in Table 1, the majority of patients received Tacrolimus based IS (87%).

**Table 1:** Demographic and clinical characteristics of the study population.

	**Treatment group**					*P*-value^*^
	**All patients (*N =* 91)**	IS reduction^*^ (*N =* 53)	Leflunomide^*^ (*N =* 30)	**IVIG (*N =* 5)**	**Cidofovir (*N =* 3)**	
Male sex, *N* (%)	63 (69)	39 (74)	21 (70)	1 (20)	2 (67)	0.80
Deceased donor, *N* (%)	74 (81)	43 (81)	23 (77)	0 (0)	3 (100)	0.78
ABO incompatible TX *N* (%)	5 (5.5)	3 (5.7)	2 (6.7)	0 (0)	0 (0)	>0.99
Age at transplantation (years), mean ± SD	51 ± 15	53 ± 16	50 ± 15	58 ± 9	43 ± 7	0.39
Cold ischemia time (h), mean ± SD	12 ± 8	11 ± 7	14 ± 10	11 ± 8	13 ± 6	0.17
HLA Mismatch, median (IQR)	3 (2–4)	3 (2–4)	3 (1–4)	4 (2)	2 (2–4)	0.48
Sensitized, *N* (%)	15 (16)	10 (19)	5 (17)	0 (0)	0 (0)	>0.99
Donor age in years, median (IQR)	55 (46–67)	57 (48–66)	52.5 (43–71)	54 (31–76)	46 (27–46)	0.98
eGFR three months after Tx, median (IQR), ml/min/1.73 m^2^	42.5 (32.9–54.5)	48.4 (35.8–56.7)	40.6 (32.6–50.1)	28.0 (17.7–41.9)	58.9 (41.5–58.9)	0.09
**Maintenance IS**						
Tacrolimus, *N* (%)	77 (85)	44 (83)	27 (90)	5 (100)	1 (33)	>0.99
CyA, *N* (%)	6 (7)	4 (8)	1 (3)	0 (0)	1 (33)	0.60
Belatacept, *N* (%)	1 (1)	1 (2)	0 (0)	0 (0)	0 (0)	>0.99
Sirolimus, *N* (%)	4 (4)	1 (2)	2 (7)	0 (0)	1 (33)	0.29
**Induction IS**, ***N*** **(%)**	60 (66)	39 (74)	20 (61)	1 (20)	0 (0)	0.36
IL-2 Antibodies, *N* (%)	40 (44)	27 (51)	13 (39)	0 (0)	0 (0)	ND
CD20 Antibodies, *N* (%)	4 (4)	2 (4)	2 (7)	0 (0)	0 (0)	ND
Apheresis, *N* (%)	15 (16)	10 (19)	5 (17)	0 (0)	0 (0)	ND
Depleting antibodies, *N* (%)	12 (13)	6 (11)	3 (10)	2 (40)	1 (33)	ND

*TX, Transplantation; HLA, human leukocyte antigen, sensitized: latest CDC PRA > 40% and/or donor specific antibody levels MFI > 1,000 (34); eGFR, estimated glomerular filtration rate; IS, immunosuppression; CyA, Cyclosporin A; d, days; IL, interleukin; ND, not done; SD, standard deviation; IQR, Interquartile range*,

* *p was calculated for comparison of the IS-reduction and Leflunomide group; intravenous immunoglobulins (IVIG) and cidofovir group were not compared due to low sample size; t-test for normally distributed continuous data, Mann–Whitney U-test for analysis without normal distribution, Fischer's exact test for categorized data*.

Induction IS was administered in 60 patients, most frequently Basiliximab (34/57%). Depleting antibodies were administered in 12 (14%) patients. Median HLA-mismatch was 3 (IQR 2–4), and median donor age was 55 (IQR 46–66.5) years. Graft function measured by the CKD-EPI formula 3 months after TX was 43 (IQR 33–55) ml/min/1.73 m^2^.

### Treatment Groups

**Figure 2:**
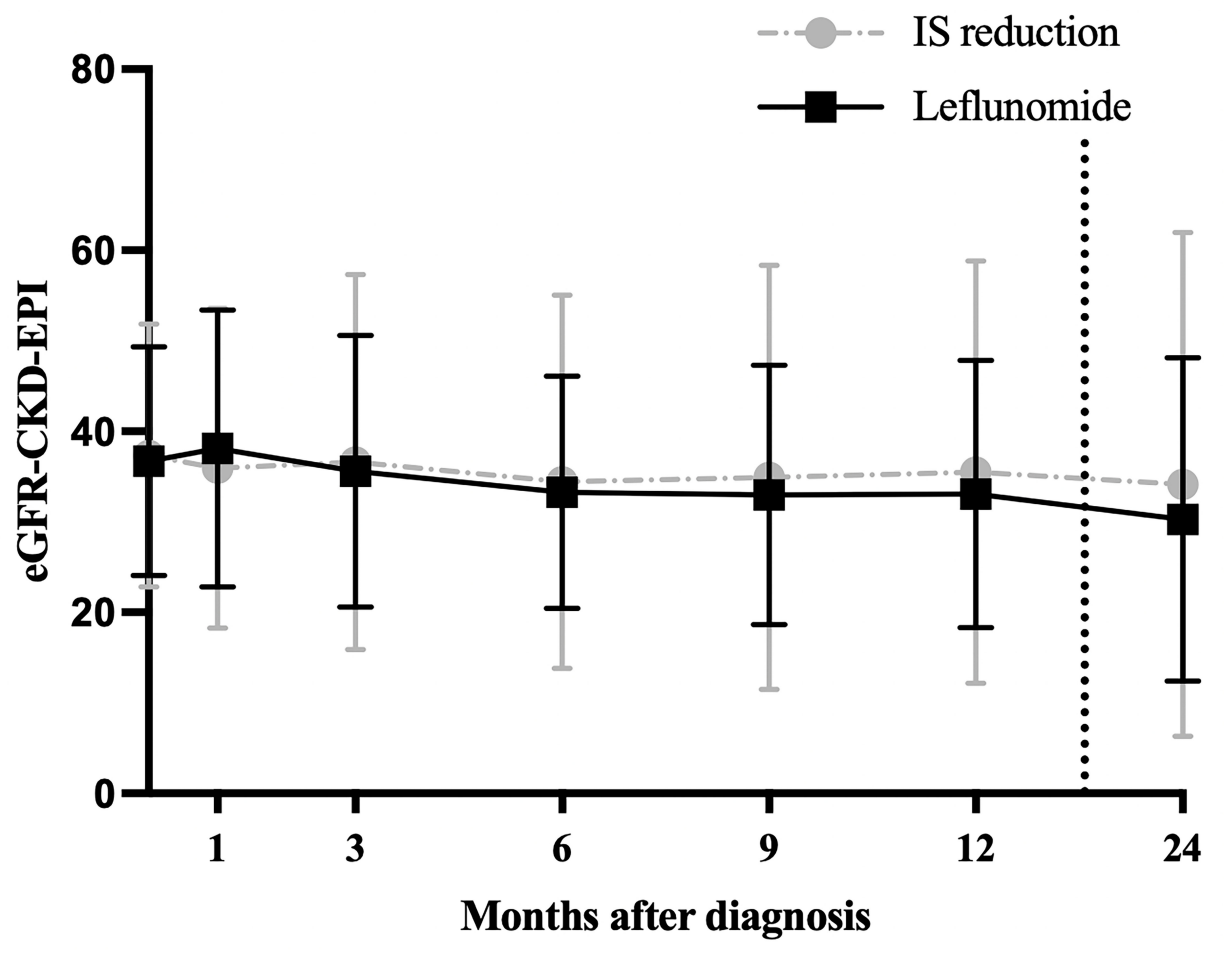
Graft function in the 24 months after biopsy; shows the eGFR in ml/min/1.73 m^2^ measured by the CKD-EPI equation between two major therapy groups. IS: immunosuppression reduction vs. Leflunomide. Dotted line between months 12 and 24 was used for visualization purposes.

Since treatment strategies may further influence outcomes, patients were divided into four groups, according to the PyVAN treatment strategy. In this study, 53 (58%) patients underwent standardized reduction of immunosuppression (details as shown in methods section), 30 (33%) patients were switched from MMF/Azathioprine to Leflunomide. Furthermore, five (6%) patients received IVIG and three (3%) patients Cidofovir as rescue medications. Table 1 displays the demographic- and baseline clinical parameters of all 91 study participants. Baseline characteristics did not differ significantly between two major treatment groups (IS-reduction vs. Leflunomide). Due to the small sample size in the Cidofovir and IVIG therapy groups, those patients were not included in further analyses. Graft function measured by the CKD-EPI equation did not differ significantly between the groups over the course of the first 12 months and after 24 months (Figure 2). The levels of primary IS are displayed in a **Table 3**.

### BK Viremia

Median time to the first detection of any positive BK viremia was 113 (IQR 81–215) days after TX, median time to biopsy proven PyVAN was 181 (IQR 125–317) days. The median viral load at the time of index biopsy was 2.15E+04 (IQR 1.70E+03–1.77E+05) copies/ml and median peak viremia was 3.6E+04 (IQR 2.7E+03–3.3E+05) copies/ml.

Neither the timing of first positive BK viremia nor the maximum BK viral load differed significantly between patients in the IS reduction group and patients receiving Leflunomide at any timepoint (Table 2). About 40% of patients achieved complete BK virus clearance during the observation time. Patients treated with Leflunomide showed higher rates of complete virus clearance at the last follow-up or after 24 months compared with patients in the IS reduction group (57 vs. 32%; *p* = 0.03).

**Table 2:** Polyomavirus associated nephropathy (PyVAN)-associated data of the study population.

	**Treatment group**					*P*-value^*^
	**All patients** **(*N =* 91)**	IS reduction^*^ **(*N =* 53)**	Leflunomide^*^ **(*N =* 30)**	**IVIG** **(*N =* 5)**	**Cidofovir** **(*N =* 3)**	
Time to first positive PCR in serum (d) median (IQR)	113 (81–215)	113 (85–243)	106 (77–178)	92 (18–143)	119 (181–277)	0.84
Days until PyVAN, median (IQR)	181 (125–317)	175 (122–347)	185.5 (130.2–334)	343 (109–353)	192 (122–222)	0.60
eGFR at PyVAN diagnosis, mean ± SD	36 ± 14	37 ± 15	37 ± 13	20 ± 16	38.0 ± 14	0.67
BK viremia at PyVAN diagnosis, median (IQR); copies/ml	2.1E+04 (1.7E+03–1.8E+05)	9.6E+03 (1.6E+03–9.2E+04)	8.7E+04 (1.6E+03–3.2E+05)	9.3E+05 (2.8E+04–1.9E+08)	1.0E+04 (4.6E+02–3.4E+04)	0.14
Max. BKV load, median (IQR); copies/ml	3.6E+04 (2.7E+03–3.3E+05)	2.4E+04 (1.9E+03–1.9E+05)	8.70E+04 (5.10E+03–6.0E+05)	9.3E+05 (2.0E+05–7.6E+07)	1.0E+04 (4.6E+02–3.4E+04)	0.43
Rejection at the time of PyVAN diagnosis *N* (%)	29 (32)	14 (27)	9 (30)	3 (60)	3 (100)	0.61
ABMR *N* (%)	2 (2)	2 (4)	0 (0)	0	0 (0)	ND
TCMR *N* (%)	27 (29)	12 (23)	9 (30)	3 (60)	3 (100)	0.46
Rejection before PyVAN diagnosis *N* (%)	30 (33)	16 (30)	8 (27)	1 (20)	3 (100)	>0.99
Rejection after PyVAN diagnosis *N* (%)	17 (18.7)	8 (15)	7 (23)	0	2 (33)	0.71

*PCR, polymerase chain reaction; PyVAN, BK polyomavirus nephropathy; BKV, BK-Virus; eGFR, estimated glomerular filtration rate according to the CKD-EPI equation; ABMR, antibody mediated rejection; TCMR, T-cell mediated rejection; ND, not done; SD, standard deviation; IQR, interquartile range*,

* *p refers to a comparison of patients in the IS reduction and patients in the Leflunomide group; t-test for normally distributed continuous data, Mann–Whitney U-test for analysis without normal distribution, Fischer's exact test for categorized data*.

**Table 3:** Level of calcineurin–inhibitors after the diagnosis of BK (PyVAN).

**Primary immunosuppression after diagnosis of PyVAN**		
**Month after diagnosis**	**Tacrolimus**	**Cyclosporin A**
0	7.04 ± 2.77	74.54 ± 53.49
1	7.22 ± 2.80	127.20 ± 71.63
3	6.20 ± 2.19	105.15 ± 75.97
6	6.13 ± 2.06	43.67 ± 27.46
9	6.06 ± 2.09	62.50 ± 63.05
12	5.79 ± 2.26	35.57 ± 22.70
24	5.76 ± 2.35	56.00 ± 24.04

*Values displayed as trough levels, expressed as mean ± SD in ng/ml. PyVAN, polyomavirus nephropathy*.

### Graft Survival and Viral Load Kinetics

As shown in Table 4, baseline data were not significantly different in subjects with and without graft loss. Moreover, there was no relation regarding diagnosis period (years 2001–2006, 2007–2012, and 2013–2018) and the observed graft loss frequency rate. The overall frequency of rejections in the index biopsy was comparable in the groups with and without graft loss (35 vs. 31%). Neither the frequency of concomitant TCMR (29.6 vs. 30%), nor antibody mediated rejection (5 vs. 1.4%) were significantly related to graft loss in this cohort of PyVAN subjects.

**Table 4:** Transplant and demographic patient characteristics in relation to a graft loss.

	**All patients *N =* 91**	**Graft loss *N =* 20**	**No graft loss *N =* 71**	*P*-value^*^
Donor age mean ± SD	54.8 ± 16.5	51.1 ± 19.1	56.0 ± 15.6	0.30
Recipient age mean ± SD	51.4 ± 15.3	51.4 ± 13.7	51.5 ± 15.8	0.97
Deceased donor *N* (%)	74 (81)	16 (80)	58 (82)	>0.99
Time to diagnosis (days) median/IQR	181 (125–317)	184 (106–241)	181 (129–381)	0.47
Cold ischemia time (h) mean ± SD	12.2 ± 8.2	13.1 ± 8.1	12.2 ± 8.3	0.64
HLA–Mismatch (total) median/IQR	3 (2–4)	2 (3.5–4.5)	3 (2–4)	0.29
CMV recipient status neg/pos *N* (%)	32 (37)	5 (25)	27 (40)	0.29
CMV donor status neg/pos *N* (%)	33 (39)	8 (45)	25 (38)	0.79
**Graft function after KTX, CKD–EPI–eGFR, ml/min/1.73 m** ^**2**^				
Graft function at index biopsy, mean ± SD	37 ± 14	28 ± 10	38 ± 15	0.08
Graft function 3 months after index biopsy, mean ± SD	45 ± 18	42 ± 17	46 ± 18	0.55
Viral load at the index biopsy copies/ml, median (IQR)	2.15E+04 (1.70E+03–1.77E+05)	1.20E+05 (1.00E+03–5.50E+05)	2.00E+04 (1.70E+03–1.20E+05)	0.30
**Biopsy findings in index biopsy**				
Interstitial fibrosis (ci) mean/SD	1.39 (0.98)	1.40 (0.94)	1.39 (1.00)	0.96
Tubular atrophy (ct) mean/SD	1.07 (0.86)	1.30 (0.92)	1.00 (0.83)	0.17
Total inflamation (ti) mean/SD	1.64 (0.89)	1.38 (0.92)	1.69 (0.89)	0.37
**Diagnosis period**				0.10
2001–2006 *N* (%)	19 (21)	4 (21)	15 (79)	ND
2007–2012 *N* (%)	37 (41)	12 (32)	25 (68)	ND
2013–2018 *N* (%)	35 (38)	4 (11)	31 (89)	ND

*HLA, human leukocyte antigen; CMV, cytomegalovirus; ND, not done; KTX, kidney transplantation; SD, standard deviation; IQR, Interquartile range; IVIG, intravenous immunoglobulins. T-test for normally distributed continuous data, Mann -Whitney U-test for analysis without normal distribution, Fischer's exact test for categorized data*.

* *p refers to a comparison of patients with and without graft loss*.

During the follow-up period of 24 months after biopsy, death-censored graft loss occurred in 20 (22%) patients within 350 ± 240 days. The incidence rate for graft loss was 1 per 100 patient months. While baseline virological variables were comparable between patients with and without graft loss (Table 4), we observed that patients with graft loss did not have a significant drop of viremia between baseline viremia and viremia at months 3, (*p* = 0.9), 6 (*p* = 0.5), and 12 (*p* = 0.25). In contrast, patients without graft loss experienced a highly significant drop of absolute viremia between baseline and months 3 (*p* < 0.001), 6 (*p* < 0.001), and 12 (*p* < 0.001).

### Multivariable Poisson Model

**Figure 3:**
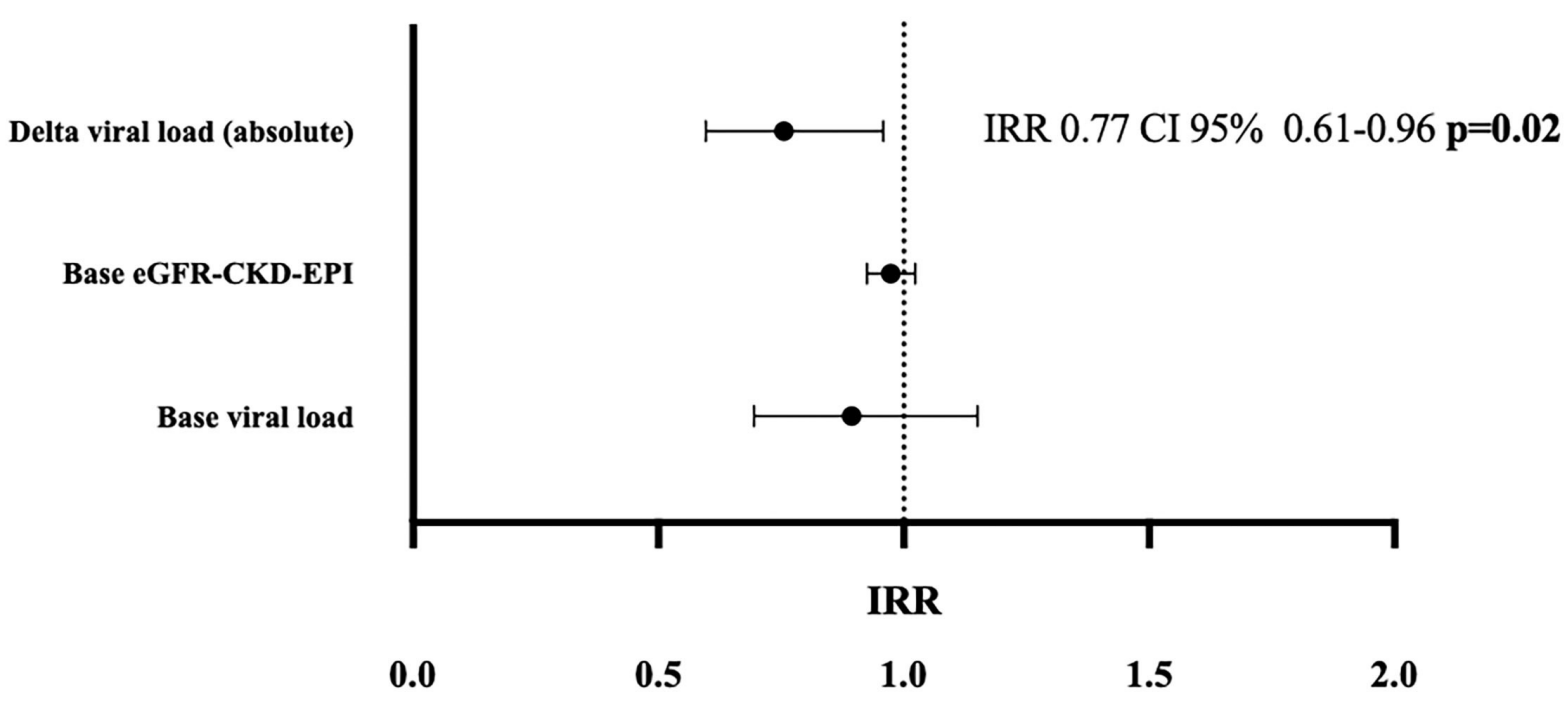
Multivariable Poisson regression model; shows the incidence rate ratios (*IRR*s) for death censored graft loss after 2 years for absolute viral load changes corrected for the baseline eGFR and baseline viral load at biopsy. Absolute delta viral load over 24 months was assessed between months 0–1, 1–3, 3–6, 6–12, and 12–24. Poisson models for estimation of *IRR* with 95% *CI*s; eGFR-CKD-EPI: estimated glomerular filtration rate measured by the CKD-EPI equation, base viral load: BK-Virus viral load in plasma at the time of diagnosis.

To further model the complex interplay of exposure time and absolute and relative viral load kinetics as risk factors for death-censored graft loss, we applied a multivariable Poisson model. This allowed for correction for multiple variables at distinct time points in relation to exposure time (time intervals: months 0–1,1–3,3–6,6–12, and 12–24). The absolute viral load change was a significant risk factor for graft survival (*IRR* = 0.78 95% *CI* 0.64–0.97, *p* = 0.03), showing that each log unit drop in absolute viral load decreased the risk for graft loss by ~22%. *IRR* was not different among treatment groups (IS reduction vs. Leflunomide 0.05 vs. 0.04) and was therefore not included in the multivariable model. Even after correction for baseline viral load and baseline eGFR, the absolute viral load change remained an independent protective factor for graft loss (*IRR* = 0.77, 95% *CI* 0.61–0.96, *p* = 0.02) (Figure 3). In contrast to absolute viral load changes, relative viral load changes were not significantly associated with graft loss (*IRR* = 0.98 95% *CI* 0.97–1.00, *p* = 0.1). Neither Tacrolimus- nor Cyclosporin A categories of through levels at each timepoint after diagnosis were significantly associated with graft loss [*IRR*: categories low (1.7), medium (0.8), and high (1.9); *p* = 0.5].

### Histological Findings

**Figure 4:**
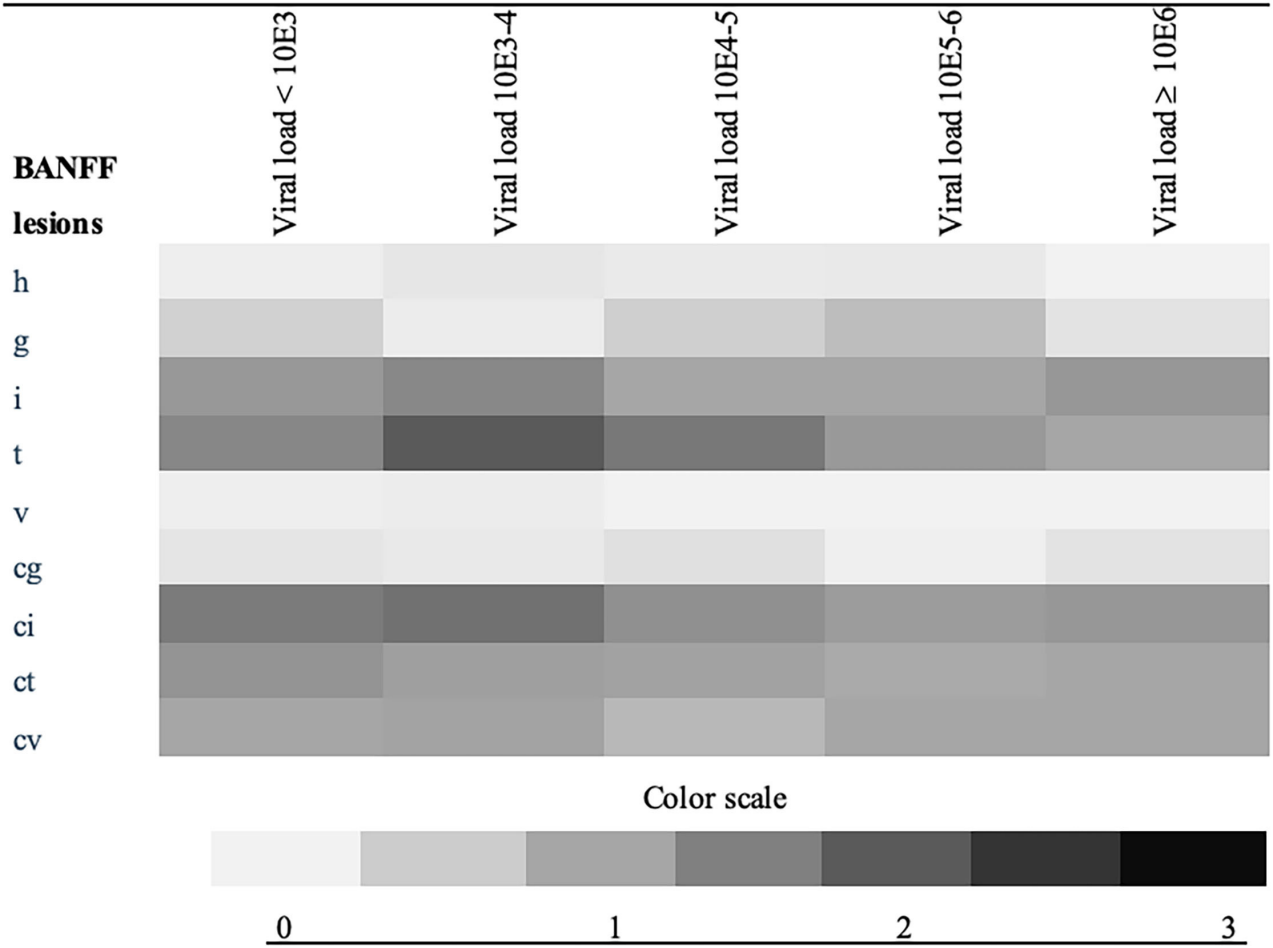
Heat map of BANFF single lesions in the index biopsy in relation to absolute viral load; shows the mean value of BANFF scores (color scaled) at the index biopsy according to the viral load at the time of the diagnosis, (copies/ml), h, arteriolar hyalinosis; g, glomerulitis; i, interstitial inflammation; v, intimal arteritis; t, tubulitis; cg, transplant glomerulopathy; ci, interstitial fibrosis; ct, tubular atrophy; cv, arterial fibrous intimal thickening, color scale describes the mean score of each group.

We analyzed the relationship between absolute viral loads at time of diagnosis with biopsy findings: Acute and chronic histological lesions assessed according to BANFF criteria at the time of diagnosis in relation to the absolute viral load at biopsy are displayed in form of a heat map in Figure 4. The BANFF single lesions did not differ significantly between patients with and without later graft loss. Furthermore, the extent of chronic injury reflected by BANFF lesion scores interstitial fibrosis (ci) and tubular atrophy showed no correlation with graft loss (Table 4). Concomitant rejection event rate was similar between the two major therapy groups (Table 2).

## Discussion

In this large retrospective analysis of a detailly characterized cohort of PyVAN patients, we demonstrate the clinical relevance of absolute BKPyV kinetics as an important prognostic marker for graft loss. Absolute (and not relative) BK viral load reductions were associated with a significantly decreased graft loss rate of 22% per log unit change after adjustment for other established risk factors. Our findings suggest that in clinical routine, attention should not only be given to patients with high viral load or chronic lesions at the index biopsy but also on patients with an insufficient reduction of viremia over time.

Our findings are partly in line with two prior studies by Nickeleit et al. (16, 35), demonstrating associations with worse graft function and high plasma viral load at index biopsy. In addition, we observed that patients with graft loss had a significantly worse graft function already at the time of index biopsy, however, after correction in a time adjusted multivariable model, this finding lost statistical significance. In most of the studies, only a minority of patients reached complete viral clearance (copies/ml below detection limit), a parameter therefore largely unsuitable for treatment surveillance (16–18). In contrast, assessing absolute viral load changes during the whole course of disease may provide an important clinical tool.

In our study, neither BANFF scores for ci or ct nor total inflammation differed significantly between patients with and without graft loss. These findings may be somewhat discrepant to a prior multicenter study (29, 30). We believe that these discrepancies are potentially related to a different study approach (static vs. dynamic) and the previously described problem of sampling errors (17) in PyVAN, which show the limitation of cross-sectionally assessed parameters, such as biopsies and support the use of dynamic assessments like absolute viral load changes for clinical management of PyVAN patients.

There is some evidence that the cumulative viremia is associated with unfavorable outcomes (15, 21). In contrast, a smaller study by Simard-Meilleur et al. demonstrated that the absolute viremia alone was not associated with further eGFR loss in a cohort of patients with PyVAN or significant BK viremia (36). Notably, in contrast with the cited studies, our analysis was focused on patients with biopsy-proven PyVAN only—a patient group commonly underrepresented in prior studies.

The studies analyzing alternative treatment regimens included mostly small sample sizes (21, 23–26, 28, 29). Besides, in our comparably large study we did not find significantly improved graft survival in patients receiving a specific treatment regimen. However, patients under Leflunomide showed higher rates of complete viral clearance, a supposed surrogate for treatment success, which is in line with a recent systematic review (24). The observed viral clearance in our study of 60% suggests that in selected patients, i.e., without relevant change in absolute viremia, Leflunomide may be considered as a valid alternative treatment option.

There is some evidence that concomitant rejection impacts graft survival in patients with PyVAN (2, 37). However, the discrimination of interstitial inflammation/tubulitis and attribution to concurrent TCMR vs. resolving PyVAN is a remaining clinical challenge and not fully resolvable in renal transplant pathology due to technical aspects (18). In our cohort, however, we did not find a higher frequency of concomitant rejections at index biopsy in patients with graft loss, excluding the possibility that concomitant rejection may have biased our findings. This is in line with a prior study where rejection at index biopsy was not associated with graft loss rates (38).

The use of Tacrolimus compared with CyA is a well-described risk factor for PyVAN (3, 10, 39–41). We assessed semi-quantitatively the cumulative level of CNI based IS by including all serial measurements of CNI levels in a Poisson model. We observed that the degree of CNI based IS after PyVAN diagnosis did not differ between patients with or without graft loss. This suggests that the short-term prognosis of PyVAN patients is more determined by absolute viral kinetics than the level of CNI based IS.

The current study has multiple strengths, most importantly the large sample size and serial assessment of multiple parameters allowing for their inclusion into a longitudinal Poisson model and adjustment of our results to time of exposure. Moreover, this study focused on purely biopsy proven PyVAN, while previous studies have included presumptive and proven PyVAN in variable proportions.

The major inherent limitations of retrospective studies are also applicable to the current study. While the decision for treatment allocation was individual, treatment groups were well-balanced regarding the baseline clinical- and virological risk factors arguing against a treatment related bias. Moreover, a delay in diagnosis and treatment is unlikely as time to first positive viremia was similar between groups. While our analysis covers a large study period of 18 years, we did not find a significantly higher graft loss rate in relation to the timing of diagnosis arguing against a relevant time-related bias. Further, study inclusion was based on histological diagnosis of PyVAN, therefore, mostly excluding a strong selection bias.

In conclusion, this study provides evidence for the prognostic importance of absolute BK viremia kinetics as a dynamic parameter indicating short-term graft survival independently of other established risk factors. Our findings support serial measurement of absolute BK viremia load changes to early identify patients with persistent viremia levels and consequently higher risk for graft loss.

## Data Availability Statement

The raw data supporting the conclusions of this article will be made available by the authors, without undue reservation.

## Ethics Statement

The studies involving human participants were reviewed and approved by Medical University of Vienna Institutional Review Board (Nr.1291/2020). Written informed consent for participation was not required for this study in accordance with the national legislation and the institutional requirements.

## Author Contributions

HO, ME, and ŽK participated in the research design, performance of the research, data analysis, interpretation of results, and writing of the manuscript. HH, JK, SA, TS, GB, KH, BW, JW, RS, and LW participated in performance of research, data analysis, and writing of the manuscript. All authors contributed to the article and approved the submitted version.

## Funding

Funding for this study was obtained by the Medical Scientific Fund of the Mayor of the City of Vienna (project number 19016), Vienna, Austria.

## Conflict of Interest

The authors declare that the research was conducted in the absence of any commercial or financial relationships that could be construed as a potential conflict of interest.

## Publisher's Note

All claims expressed in this article are solely those of the authors and do not necessarily represent those of their affiliated organizations, or those of the publisher, the editors and the reviewers. Any product that may be evaluated in this article, or claim that may be made by its manufacturer, is not guaranteed or endorsed by the publisher.

## Abbreviations

AZA: Azathioprine
BKPyV: BK polyomavirus
CNI: calcineurin inhibitor
CyA: Cyclosporin A
HLA: human leukocyte antigen
IS: immunosuppression
KTX: kidney transplantation
IVIG: intravenous immunoglobulins
MMF: mycophenolate mofetil
PCR: polymerase chain reaction
PyVAN: polyomavirus associated nephropathy
SV40: simian virus 40
TCMR: T-cell-mediated rejection
TX: transplantation.
